# Effects of gut microbiota on prostatic cancer: a two-sample Mendelian randomization study

**DOI:** 10.3389/fmicb.2023.1250369

**Published:** 2023-11-06

**Authors:** Qingpeng Xie, Bin Hu

**Affiliations:** Department of Urology, Cancer Hospital of China Medical University, Liaoning Cancer Hospital and Institute, Shenyang, Liaoning, China

**Keywords:** gut microbiota, prostatic cancer, Mendelian randomization study, endometriosis, causal inference

## Abstract

**Aim:**

Recent observational and small-sample case-control studies have shown a relationship between gut microbiota composition and prostatic cancer (PCa). Nevertheless, the causal association between gut microbiota and PCa is still unclear. Herein, we used the Mendelian randomization (MR) method to explore the potential causal relationship between gut microbiota and PCa.

**Methods:**

In this two-sample MR study, data were extracted from the summary statistics of gut microbiota from the largest available genome-wide association study meta-analysis conducted by the MiBioGen consortium (*n* = 14,306) and the Dutch Microbiome Project (*n* = 8,208). Summary statistics for PCa were obtained from the FinnGen consortium release data (*n* = 95,213). Inverse variance weighted (IVW), MR-Egger, strength test (F), and MR-PRESSO were used to examine the potential causal association between gut microbiota and PCa. Cochran's Q statistics were used to quantify the heterogeneity of instrumental variables.

**Results:**

IVW estimates suggested that the relative abundance of *Akkermansia muciniphila* (odds ratio [OR] = 0.7926, 95% confidence interval [CI]: 0.6655–0.9440) and *Bacteroides salyersiae* (OR = 0.9023, 95% CI: 0.8262–0.9853) were negatively associated with the odds of PCa, while that of *Eubacterium biforme* (OR = 1.1629, 95% CI: 1.0110–1.3376) was positively associated with the odds of PCa. In addition, we explored these relationships among patients without other cancers and similarly found that the relative abundance of *Akkermansia muciniphila, Bacteroides salyersiae*, and *Eubacterium biforme* were linked to PCa (all *P* < 0.05).

**Conclusion:**

Gut microbiota potentially influenced the occurrence of PCa. Our findings may provide some new ideas for researching the methods of PCa prevention. In addition, further studies are needed to explore the causal association and specific underlying mechanisms between gut microbiota and PCa.

## Introduction

The role of gut microbiota in prostate cancer (PCa) has received a lot of attention in recent years (Rizzo et al., 2022), and the concept of “gut-prostate axis” has been proposed (Fujita et al., 2023; Matsushita et al., 2023). Previous basic studies suggested that the underlying mechanisms of gut microbiota's effects on the incidence and progression of PCa may involve modulating inflammation, oxidative stress, and immune function and interfering with lipid metabolism (Porter et al., 2018; Wheeler and Liss, 2019). Nevertheless, evidence from population-based studies on the relationship between gut microbiota and PCa remains limited, and most of the existing studies are small-sample case-control studies.

Studies have suggested that patients with PCa exhibit an increased relative abundance of the bacterial genera *Veillonella, Bacteroides* (Alanee et al., 2019), *Streptococcus* (Liss et al., 2018), *Rikenellaceae, Alistipes*, and *Lachnospira* (Matsushita et al., 2021). While in control groups, the relative abundance of *Faecalibacterium prausnitzii* and *Eubacterium rectalie* was higher than that in cases (Golombos et al., 2018). However, the firm conclusions for the causal relationship and its direction between gut microbiota and PCa are not yet enough to draw according to the existing observational studies' evidence. Inherent defects limited traditional observational studies, and thus the causal role of gut microbiota in the risk of PCa remained to be clarified (Tong et al., 2020). In spite of the fact that randomized controlled trial is recognized as a gold standard for determining causality, its application in clinical settings is impractical due to the long incubation period from certain microbiota exposure to oncogenesis (Spieth et al., 2016). Under the circumstances, a novel method for investigating the causal association between gut microbiota and PCa is warranted.

Mendelian randomization (MR) analysis has become a widely used approach that exploits single nucleotide polymorphisms (SNPs) as unconfounded instrumental variants (IVs) to explore the potential causal relationships between environmental exposures and diseases (Davey Smith and Hemani, 2014; Sekula et al., 2016). MR can avoid reverse causality inferences and reflect the long-term effects of exposures on outcomes. A recent two-sample MR analysis assessed the causal effect of gut microbiota on cancer risk and showed that the relative abundance of *Alphaproteobacteria, Rhodospirillales, Adlercreutzia*, and *Coprobacter* was associated with PCa (Wei et al., 2023). However, the findings of Wei's study were not in accordance with those of other epidemiological studies, and they also did not explore the causal associations between gut microbiota and cancer risk at the species level.

Herein, in this two-sample MR study, we aimed to investigate the potential causal relationship between gut microbiota at different levels, especially at the species level, and PCa in order to provide some new ideas for exploring the methods for PCa prevention and treatment.

## Methods

### Data sources

**Figure 1:**
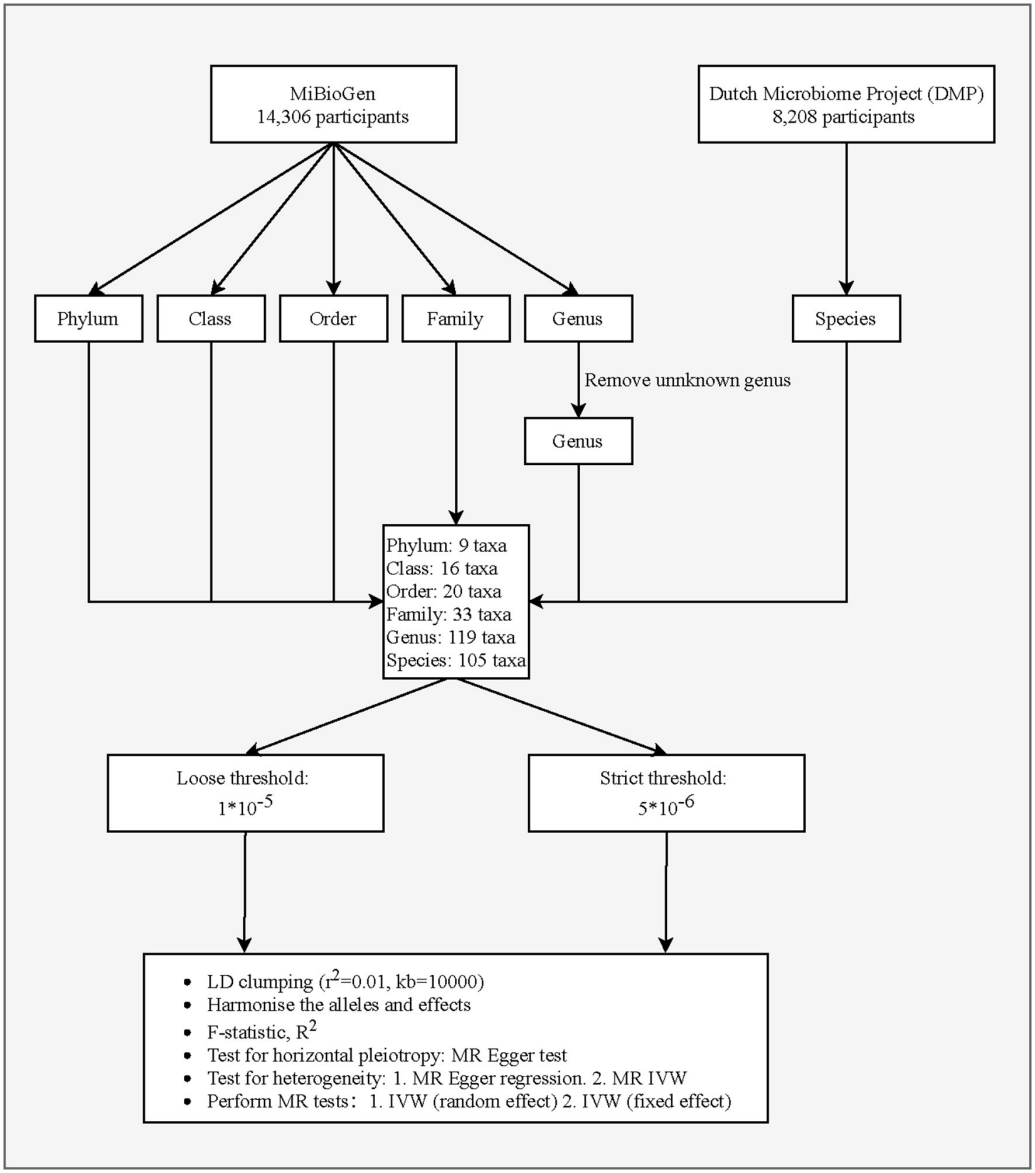
Flowchart of the study design.

This study is a two-sample MR analysis. Data from the genome-wide association studies (GWASs) were extracted for gut microbiota and PCa. Figure 1 is the flowchart of this research procedure. Genetic variants of the gut microbiota were obtained from MiBioGen (Kurilshikov et al., 2021) and the Dutch Microbiome Project (DMP) (Lopera-Maya et al., 2022), and the sample size was 14,306 and 8,208, respectively. PCa cases (*n* = 6,311) and controls (*n* = 88,902) were obtained from the FinnGen consortium initially (Kurki et al., 2023). The detailed descriptions of exposure and outcome, including the data source, microbial taxa, race of population, sample size, the total number of SNPs, and website information, are presented in Table 1.

**Table 1:** Information of the data source for gut microbiota and PCa.

**Variables**	**Consortium**	**Traits**	**Year**	**Population**	**Sample size**	**nSNPs**	**nTaxa**	**Websites**
Exposure	MiBioGen	Phylum	2021	European	14,306		9	https://mibiogen.gcc.rug.nl/menu/main/home
		Class	2021	European	14,306		16	
		Order	2021	European	14,306		20	
		Family	2021	European	14,306		33	
		Genus	2021	European	14,306		119	
	DMP	Species	2021	European	8,208		105	https://dutchmicrobiomeproject.molgeniscloud.org/menu/main/home
Outcome	FinnGen	PCa	2021	European	95,213	16,378,835	-	https://www.finngen.fi/en

PCa, prostatic cancer; SNP, single nucleotide polymorphism; DMP, Dutch Microbiome Project.

The study data from the databases are de-identified and publicly available. The informed consent of all participants (legal guardians for participants under 18 years old) has been obtained in each GWAS involved in this study and was ethically approved by the respective institutions. Therefore, no ethical approval of our agency's institutional review board was required.

### Single nucleotide polymorphism selection

We extracted six levels of gut microbiota taxa, including phylum, class, order, family, genus, and species. SNPs significantly associated with gut microbiota were selected as potential instrument variables (IVs). We used two threshold standards to select the IVs, including a loose threshold (*P* < 1.0 × 10^−5^) and a strict threshold (*P* < 5.0 × 10^−6^). SNPs with a minor allele frequency of ≤ 0.01 were removed. The linkage disequilibrium threshold was set to be r^2^ = 0.01, clumping distance = 10,000 kb (for loose threshold), and r^2^ = 0.001 clumping distance = 10,000 kb (for strict threshold), respectively. We applied the MR-Egger regression test to monitor the potential horizontal pleiotropic effect (Burgess and Thompson, 2017), namely the confounding effect resulting from other diseases, which may violate the second assumption in MR analysis (only affect the outcome via the exposure). The intercept item of MR-Egger that was significant represents the existence of pleiotropy. In addition, palindromic SNPs were deleted due to the principle of MR to ensure that the same allele corresponds to the effects between SNPs and exposure and on the outcome.

### The assumptions of MR analysis

For the purpose of minimizing the impact of bias on the results, MR must conform to three important assumptions. First, IVs must be independent of confounders related to exposure and outcome. Second, the IVs should be significantly linked to the exposure. The association strength between gut microbiota and IVs was estimated using the formula: F = β^2^/SE^2^ (Xie et al., 2023), where β was the regression coefficient for gut microbiota and IVs and SE was the standard error. A weak association between IVs and exposure is recognized when F < 10. Third, IVs influence outcomes through exposure only; that is, there is no horizontal pleiotropic effect of IVs on outcomes.

### Statistical analysis

The statistical analyses were performed by R version 4.2.0 (Institute for Statistics and Mathematics, Vienna, Austria). The R package “TwoSampleMR” was used for MR analysis of the causal association between gut microbiota and PCs. *P* < 0.05 indicates the statistical significance of evidence for a potential causal relationship. The Wald ratio method was utilized to assess the role of individual IVs in the causal estimates. Calculation for the causal effect values was done using the inverse variance weighted (IVW) test, which is the primary method to obtain unbiased estimates when horizontal pleiotropy is absent. We used both fixed and random effects models for the IVW test. The effect size was expressed by odds ratios (ORs) with 95% confidence intervals (CIs). In addition, Bonferroni thresholds were also utilized to adjust for the *P*-value to control for multiple tests.

The test for heterogeneity was Cochrane's Q test, and IVs *P* < 0.05 were recognized as heterogeneous. The intercept of MR-Egger regression examined the potential pleiotropy in IVs, and *P* > 0.05 was deemed to be no horizontal pleiotropy. The examination of possible outliers was done using the MR-Pleiotropy RESidual Sum and Outlier (MR-PRESSO) test (R package “MR-PRESSO”) (Verbanck et al., 2018). Moreover, we performed the reverse causality analysis between gut microbiota and PCa.

## Results

### Instrumental variables selection

After quality control, we identified 2,616 (*P* < 1.0 × 10^−5^) and 1,371 (*P* < 5.0 × 10^−6^) SNPs as IVs for 302 bacterial taxa, which comprised 9 phyla, 16 classes, 20 orders, 33 families, 119 genera, and 105 species. Then, we evaluated the horizontal pleiotropic effect at each taxa level. For both PCa and the five levels of gut microbiota, none of the IVs were outliers through the MR-PRESSO test. IVs in this analysis had no horizontal pleiotropy after removing pleiotropic SNPs identified using the MR-PRESSO outlier test and MR-Egger regression (both MR-PRESSO global test *P* > 0.05 and MR-Egger regression *P* > 0.05).

### Two-sample MR analysis

**Figure 2:**
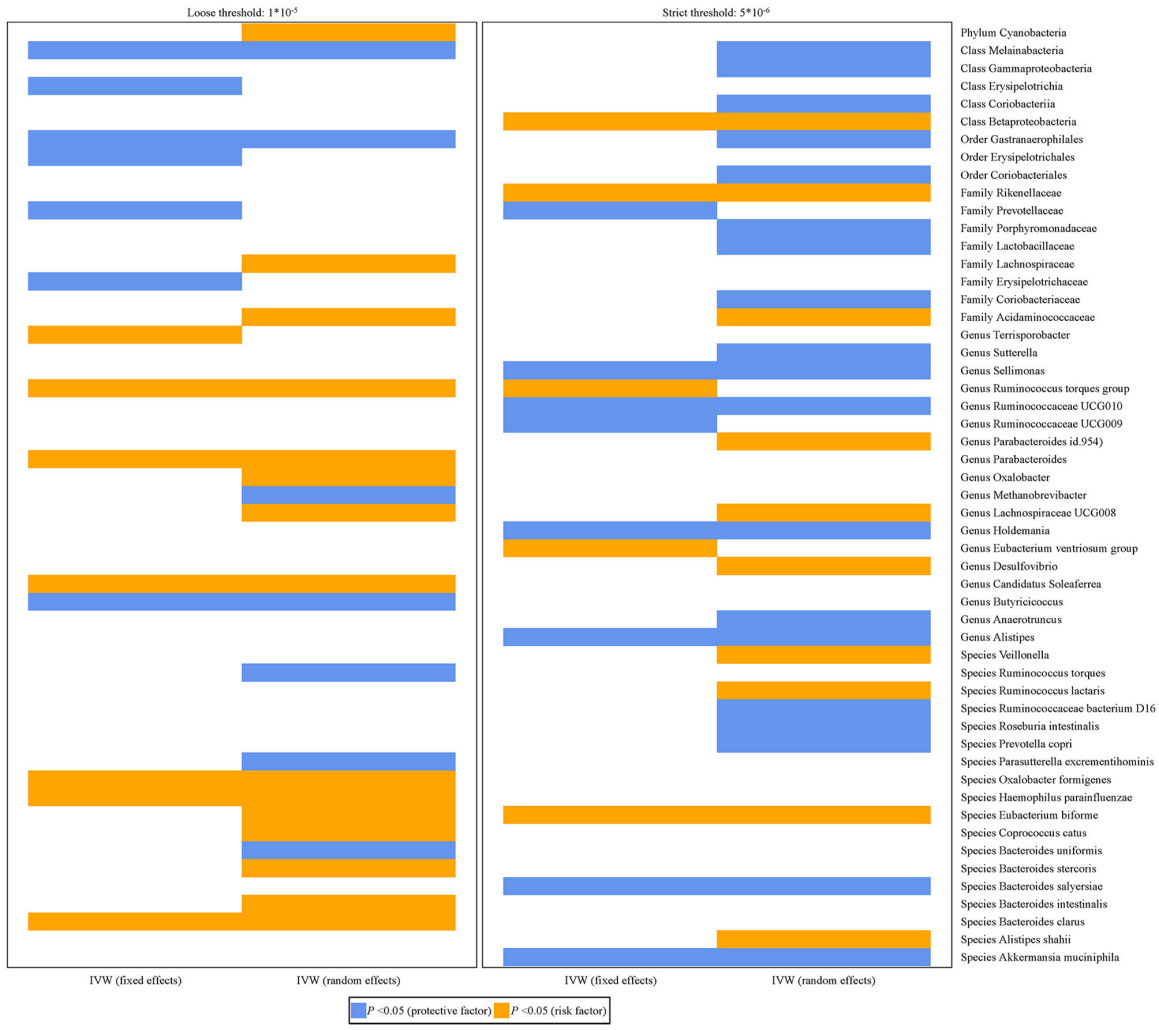
Histograms for the potential causal association between gut microbiota and PCa. The blue color represents OR < 1 while orange color represents OR > 1.

Figure 2 shows the relationship between 53 bacterial taxa and PCa. Whether the IVW estimates used a loose threshold (*P* < 1.0 × 10^−5^) or a strict threshold (*P* < 5.0 × 10^−6^), the relative abundance of seven bacterial taxa was all significantly associated with the odds of PCa. To be specific, the increased relative abundance of *Melainabacteria* (at class level), *Gastranaerophilales* (at order level), and *Prevotellaceae* (at family level) was negatively associated with the odds of PCa, while that of *Acidaminococcaceae* (at family level), *Ruminococcus torques* group, *Lachnospiraceae UCG-008* (at genus level), and *Eubacterium biforme* (at species level) had positive relationships.

When focused on the role of bacterial taxa at the species level in PCa (fixed effect), the relative abundance of *Akkermansia muciniphila* (OR = 0.7926, 95% CI: 0.6655–0.9440) and *Bacteroides salyersiae* (OR = 0.9023, 95% CI: 0.8262–0.9853) was both negatively associated with the odds of PCa, while that of *Eubacterium biforme* was positively associated with the odds of PCa (OR = 1.1629, 95% CI: 1.0110–1.3376) (Table 2).

**Table 2:** Association between gut microbiota and PCa at the species level.

**Microbiota taxa (species)**	**Pleiotropy test**		**F-statistic**	**Heterogeneity (IVW)**		**Heterogeneity (MR-Egger)**		**nSNP**	**IVW (random effect)**		**IVW (fixed effect)**		**Bonferroni threshold (0.05/nSNPs)**	**Power (%)**
	**MR-Egger intercept**	***P***		**Q**	***P***	**Q**	***P***		**OR (95% CI)**	***P***	**OR (95% CI)**	***P***		
**Malignant neoplasm of prostate**														
*Akkermansia muciniphila*	−0.0345	0.5702	167.5746	2.7913	0.5933	2.3873	0.496	5	0.7926 (0.6850–0.9172)	0.002	0.7926 (0.6655–0.9440)	0.009	0.010	0.10
*Alistipes shahii*	−0.0099	0.9119	88.61356	0.6255	0.8906	0.6098	0.7372	4	1.1745 (1.0431–1.3225)	0.008	1.1745 (0.9058–1.5230)	0.225	0.0125	0.08
*Bacteroides salyersiae*	−0.0238	0.6262	132.4007	3.5203	0.6203	3.2427	0.5181	6	0.9023 (0.8380–0.9715)	0.006	0.9023 (0.8262–0.9853)	0.022	0.008	0.06
*Eubacterium biforme*	−0.0309	0.5646	88.15534	1.4646	0.6905	0.9969	0.6075	4	1.1629 (1.0545–1.2824)	0.002	1.1629 (1.0110–1.3376)	0.035	0.0125	0.08
*Prevotella copri*	−0.0091	0.917	109.985	0.1711	0.918	0.1539	0.6948	3	0.8161 (0.7565–0.8803)	< 0.001	0.8161 (0.6299–1.0573)	0.124	0.017	0.09
*Roseburia intestinalis*	−0.0113	0.817	131.1277	0.7852	0.9404	0.7214	0.8681	5	0.8988 (0.8251–0.9792)	0.015	0.8988 (0.7409–1.0905)	0.279	0.010	0.06
*Ruminococcaceae bacterium_D16*	−0.0033	0.9555	157.0054	2.4726	0.6495	2.4689	0.4809	5	0.9143 (0.8411–0.9938)	0.035	0.9143 (0.8222–1.0166)	0.098	0.010	0.06
*Ruminococcus lactaris*	−0.005	0.9676	89.21098	0.1166	0.9434	0.114	0.7356	3	1.1357 (1.0818–1.1924)	< 0.001	1.1357 (0.9283–1.3895)	0.216	0.017	0.07
*Veillonella unclassified*	0.0328	0.4214	170.6334	1.631	0.9503	0.8646	0.9727	7	1.0663 (1.0081–1.1279)	0.025	1.0663 (0.9575–1.1875)	0.242	0.007	0.05
**Malignant neoplasm of prostate (all cancers excluded)**														
*Akkermansia muciniphila*	−0.0306	0.6265	167.5746	2.4719	0.6497	2.1799	0.5359	5	0.7857 (0.6808–0.9067)	0.001	0.7857 (0.6548–0.9428)	0.009	0.010	0.1
*Alistipes shahii*	−0.0509	0.5989	88.61356	0.9831	0.8053	0.5997	0.7409	4	1.1949 (1.0234–1.3952)	0.024	1.1949 (0.9115–1.5664)	0.197	0.0125	0.09
*Bacteroides salyersiae*	−0.0206	0.6849	132.4007	2.9379	0.7096	2.7472	0.601	6	0.9019 (0.8406–0.9677)	0.004	0.9019 (0.8228–0.9887)	0.028	0.008	0.06
*Eubacterium biforme*	−0.0404	0.4808	88.15534	1.1133	0.7739	0.3752	0.829	4	1.1876 (1.0869–1.2976)	< 0.001	1.1876 (1.0268–1.3735)	0.021	0.0125	0.09
*Prevotella copri*	−0.0493	0.6217	109.985	0.6156	0.7351	0.1588	0.6902	3	0.8608 (0.7410–0.9999)	0.049	0.8608 (0.6571–1.1277)	0.277	0.017	0.07
*Roseburia intestinalis*	−0.0088	0.8619	131.1277	0.9282	0.9205	0.8923	0.8273	5	0.9068 (0.8229–0.9993)	0.048	0.9068 (0.7412–1.1095)	0.342	0.010	0.06
*Ruminococcaceae bacterium_D16*	0.0001	0.9984	157.0054	1.0069	0.9087	1.0069	0.7996	5	0.9005 (0.8519–0.9518)	< 0.001	0.9005 (0.8063–1.0057)	0.063	0.010	0.06
*Ruminococcus lactaris*	−0.0052	0.9681	89.21098	0.2558	0.8799	0.2533	0.6148	3	1.0556 (0.9790–1.1382)	0.159	1.0556 (0.8551–1.3032)	0.614	0.017	0.05
*Veillonella unclassified*	0.0431	0.3186	170.6334	3.3993	0.7573	2.1731	0.8247	7	1.0798 (0.9926–1.1746)	0.074	1.0798 (0.9655–1.2076)	0.179	0.007	0.05

PCa, prostatic cancer; IVW, inverse variance weighted test; SNP, single nucleotide polymorphism; OR, odds ratio; CI, confidence interval. nSNP is the number of SNPs being used as IVs; F-statistic is the value of F statistics to examine the weak instrument bias; Q is the estimated effect coefficient; Malignant neoplasm of prostate marked in bold represents that participants in control group do not have PCa.

Malignant neoplasm of prostate (all cancers excluded) marked in bold represents that participants in control group do not have any cancers.

We further explored these associations among patients without any other cancers and similarly found the negative relationship between the relative abundance of *Akkermansia muciniphila* (OR = 0.7857, 95% CI: 0.6548–0.9428) and *Bacteroides salyersiae* (OR = 0.9019, 95% CI: 0.8228–0.9887) and PCa, and the positive relationship between the relative abundance of *Eubacterium biforme* (OR = 1.1876, 95% CI: 1.0268–1.3735) and PCa.

Table 2 also shows the pleiotropy and heterogeneity test results. We indicated the impact of comparatively accurate MR results in three species of gut microbiota on PCa by sensitivity analysis. No horizontal pleiotropy was observed in *Akkermansia muciniphila* (*P* = 0.5702), *Bacteroides salyersiae* (*P* = 0.6262), and *Eubacterium biforme* (*P* = 0.5646) for PCa. Furthermore, there was no heterogeneity in *Akkermansia muciniphila* (IVW: *P* = 0.5933; MR-Egger: *P* = 0.4960), *Bacteroides salyersiae* (IVW: *P* = 0.6203; MR-Egger: *P* = 0.5181), and *Eubacterium biforme* (IVW: *P* = 0.6905; MR-Egger: *P* = 0.6075) for PCa. Results of IVW were comparatively reliable when heterogeneity and pleiotropy were absent, indicating the potential causal relationships between these three species of gut microbiota and PCa were comparatively steady.

In addition, we used the Bonferroni threshold to assess these relationships and found that only high a relative abundance of *Akkermansia muciniphila* (*P* = 0.010) was associated with low odds of PCa among all participants or those without any other cancers. The results of reverse causality analysis also showed that there was no reverse causality relationship between gut microbiota and PCa (fixed effect) (Table 3).

**Table 3:** Reverse causality between gut microbiota and PCa at the species level.

**Microbiota taxa (species)**	**IVW (random effect)**		**IVW (fixed effect)**	
	**OR (95% CI)**	***P***	**OR (95% CI)**	***P***
*Akkermansia muciniphila*	0.9737 (0.8817–1.0754)	0.5995	0.9737 (0.9171–1.0339)	0.3840
*Alistipes shahii*	0.9976 (0.9518–1.0456)	0.9201	0.9976 (0.9445–1.0537)	0.9314
*Bacteroides salyersiae*	1.0747 (0.9442–1.2232)	0.2754	1.0747 (0.9494–1.2165)	0.2549
*Eubacterium biforme*	0.9954 (0.9155–1.0823)	0.9134	0.9954 (0.8982–1.1030)	0.9293
*Prevotella copri*	0.9457 (0.8978–0.9960)	0.0348	0.9457 (0.8897–1.0051)	0.0725
*Roseburia intestinalis*	0.9748 (0.9347–1.0166)	0.2327	0.9748 (0.9142–1.0393)	0.4345
*Ruminococcaceae bacterium_D16*	1.0063 (0.9027–1.1219)	0.9093	1.0063 (0.8939–1.1329)	0.9168
*Ruminococcus lactaris*	1.0157 (0.9442–1.0926)	0.6757	1.0157 (0.9444–1.0924)	0.6748
*Veillonella unclassified*	0.9995 (0.8959–1.1150)	0.9926	0.9995 (0.9073–1.1010)	0.9916

PCa, prostatic cancer; IVW, inverse variance weighted test; OR, odds ratio; CI, confidence interval.

## Discussion

We conducted an MR analysis to explore the potential causal relationship between gut microbiota and PCa. The results showed that *Melainabacteria* (class level), *Gastranaerophilales* (order level), *Prevotellaceae* (family level), *Acidaminococcaceae* (family level), *Ruminococcus torques group* (genus level), *Lachnospiraceae UCG-008* (genus level), *Akkermansia muciniphila* (species level), *Bacteroides salyersiae* (species level), and *Eubacterium biforme* (species level) were associated with PCa. To be specific, the relative abundance of *Akkermansia muciniphila* and *Bacteroides salyersiae* was both negatively associated with the odds of PCa, while that of *Eubacterium biforme* was positively associated with the odds of PCa. Besides, no reverse causality has been found between them.

The firm conclusions for the causal relationship between gut microbiota and PCa are not yet enough to draw according to the evidence from existing observational studies. In recent years, only a few studies have explored the association between gut microbiota and the risk of cancer using MR analyses, which is a widely used approach to explore the potential causal relationships between environmental exposures and diseases. For example, a univariable and multivariable MR study by Wei et al. (2023) assessed the causal effect of gut microbiota on five common cancers, including breast, endometrial, lung, ovarian, and PCa. Their results showed that a higher abundance of class *Alphaproteobacteria* was associated with a lower risk of PCa. Differently, in our study, we focused on the relationship between gut microbiota and PCa only and observed gut microbiota at phylum, class, order, family, genus, and species levels. We found that the relative abundance of *Akkermansia muciniphila, Bacteroides salyersiae*, and *Eubacterium biforme* was all associated with PCa. Our study detailed the group of gut microbiotas and found three specific species, which may provide some references for further basic and prospective research on the causal relationship between gut microbiota and PCa. In Wei's study, the associations between SNPs and PCa were obtained from the GWAS study from the Prostate Cancer Association Group to Investigate Cancer-Associated Alterations in the Genome (PRACTICAL) Consortium, which consists of cases diagnosed with PCa and controls of European descent. Another two-sample MR study by Long et al. (2023), also based on the population from the PRACTICAL Consortium, examined the causal relationship between gut microbiota and cancer. Although we used the GWAS study from the Finngen Consortium, the race of the study population was similar. Long's results showed that the genus *Ruminococcustorquesgroup*, class *Verrucomicrobiae*, family *Verrucomicrobiaceae*, order *Verrucomicrobiales*, genus *Terrisporobacter*, genus *Roseburia*, and class *Alphaproteobacteria* were causally associated with PCa. The different gut microbiota we explored may complement previous studies.

In fact, the underlying mechanisms of these gut microbiota and PCa are complex and unclear. The class *Melainabacteria* has been identified as an accurate biomarker of zinc (Zn) status in the human body (Chen et al., 2021). As Zn plays a growth-modulatory role in PCa, *Melainabacteria* may influence the occurrence and development of PCa by modulating Zn levels (To et al., 2020; Zhang et al., 2022). *Melainabacteria* exist in groundwater, wastewater treatment plants, and herbivorous mammal and human guts and have the function of synthesizing vitamins B and K, suggesting they are beneficial bacteria to their hosts (Di Rienzi et al., 2013). Similarly to the previous studies, our findings showed that an increased abundance of *Melainabacteria* was associated with low odds of PCa. *Gastranaerophilales* is one of the probiotics with impaired abundance in colitis (Wang et al., 2021; Wu et al., 2021). Inflammation is a risk factor for prostate carcinogenesis, with diet, chemical injury, and an altered microbiome being causally implicated (de Bono et al., 2020). However, no study has been conducted to describe the potential mechanisms by which *Gastranaerophilales* play a protective role in prostate carcinogenesis, which needs further basic research for clarification. *Prevotellaceae* is also a probiotic that plays an important role in colitis and some cancers (Zhang L. et al., 2019; Qu et al., 2021). Li et al. (2022) performed an analysis on alterations of gut microbiota diversity, composition, and metabonomics in benign prostatic hyperplasia rats and showed that there was a strong correlation between *Prevotellaceae* and differential metabolites. However, the specific mechanism of the potential protective role of *Prevotellaceae* in PCa has not been clear. According to previous studies, we speculated that *Prevotellaceae* may moderate inflammation (Qu et al., 2021), oxidative stress (Cui et al., 2018), and metabolic disorders (Li et al., 2022) and further influence PCa development.

The high relative abundance of the family *Acidaminococcaceae*, genus *Ruminococcus torques group*, and genus *Lachnospiraceae UCG-008* was found to be associated with a high risk of PCa in this study. *Acidaminococcaceae* clustered into one group with the *Veillonellaceae*, which has been reported as a marker of dysbacteriosis and plays a possible role in carcinogenesis (Yan et al., 2015; Kasai et al., 2016). A bidirectional, two-sample MR study on the association between Graves' disease and the gut microbiome showed that the *Ruminococcus torques group* was identified as a risk factor (Cao et al., 2023). *Ruminococcus torques group* is a genera derived from the genus *Mediterraneibacter* from the family *Lachnospiraceae* and is identified as a butyrate-producing bacterium (Salyers et al., 1977). Butyrate-producing bacteria were found to promote fat deposition because they are able to convert dietary fiber to butyrate by fermentation (Yang et al., 2010; Blaut, 2015). The abundance of *Ruminococcus torques group* is thus supposed to be positively related to fat accumulation. Excess fat can accelerate the growth of prostate tumors by inducing inflammation (Hayashi et al., 2018). In addition, altered lipid metabolism, especially the excessive accumulation of cholesterol and fatty acids, promotes the malignant transformation of PCa via the formation of cholesteryl esters (Wang et al., 2022). *Lachnospiraceae UCG-008* is considered a potential harmful genus in human colonic microbiota (Huang et al., 2019). Some medicines improve gut microbial dysbiosis by increasing beneficial bacteria and decreasing harmful bacteria, including *Lachnospiraceae UCG-008* in order to relieve diseases (Huang et al., 2019; Yin et al., 2021). In conclusion, how gut microbiota influence the development of PCa by disturbing the metabolism is still unclear; further studies are needed to clarify the specific biological mechanisms.

We additionally explored the relationships between gut microbiota and the risk of PCa at the species level. Our findings indicated that the high relative abundance of *Akkermansia muciniphila* and *Bacteroides salyersiae* was both associated with a low risk of PCa, while that of *Eubacterium biforme* was positively associated with the risk of PCa. *Akkermansia muciniphila* is a Gram-negative anaerobic bacterium that contributes to homeostasis maintenance and barrier integrity in the gastrointestinal tract (Zhai et al., 2019; Zhang T. et al., 2019). Recently, it has been reported that intravenous injection of *Akkermansia muciniphila*-derived extracellular vesicles in immune-competent mice reduced the tumor burden of PCa without inducing obvious toxicity in normal tissues, indicating a potential association between the abundance of *Akkermansia muciniphila* and PCa (Luo et al., 2021). Similarly, our MR study supplemented and suggested a potential causal relationship between the high relative abundance of *Akkermansia muciniphila* and the low risk of PCa. Wang et al. (2020) found the membrane protein from *Akkermansia muciniphila*, namely Amuc_1100, was associated with tumourigenesis of colorectal cancer through modulation of CD8 T cells in mice. Gu et al. (2021) also suggested that *Akkermansia muciniphila* and its outer protein Amuc_1100 regulated the tryptophan metabolism in colitis. Another animal study also showed the effect of improved Amuc_1100 from *Akkermansia muciniphila* on metabolism in obese and diabetic mice (Plovier et al., 2017). As the mechanism of PCa development mentioned previously, *Akkermansia muciniphila* and Amuc_1100 may also influence PCa by improving inflammation and regulating glucose metabolism and lipid metabolism, but further mechanistic exploration is needed. *Bacteroides salyersiae* is a beneficial commensal with *Akkermansia muciniphila* and plays a similar role in the host (Derosa et al., 2020). Our study considered that the application of probiotics in the prevention and treatment of PCa was very promising. Gut microflora evolves with a complex polysaccharide-rich diet and dietary fermentation, resulting in the production of short-chain fatty acids such as butyrate, which represent a primary energy source for colonic epithelial cells and preserve them from inflammation (Ahmad et al., 2000; Atarashi et al., 2013). *Eubacterium biforme* is one of them. Aside from this, a lack of fiber in diets is associated with a low concentration of short-chain fatty acids (Pituch-Zdanowska et al., 2015). Daily attention to dietary fiber consumption may be the key to preventing inflammatory diseases by regulating intestinal flora. Daily consumption of bifidobacteria-rich foods such as yogurt may be a viable way to regulate probiotics; however, whether a high relative abundance of *Eubacterium biforme* is associated with a high risk of PCa is not determinate.

As mentioned before, MR is a relatively good study design to clarify the causal effect of potential risk factors on diseases of interest. By exploring the gut microbiota that modulate PCa risk, MR studies facilitate the recommendation of public health policies and clinical interventions that effectively reduce the incidence and social burden of PCa. With the rapid development of omics technologies in recent years, including genomics, proteomics, transcriptomics, metabolomics, and epigenomics, researchers can use a large number of novel exposures/intermediate phenotypes generated in observational studies to assess associations with clinical endpoints. The MR approaches summarize the results from published GWAS studies facilitately and do not need a separate study to carry out MR analyses (Sekula et al., 2016). Compared with observational studies, which commonly suffer from biased results due to confounding, MR can deal with any confounding by design as long as a valid genetic instrumental variable is available. However, two major limitations also influence the judgment of causation through MR approaches. First, no valid instrument for every research question is available because of a lack of knowledge about MR, and the publicly available sources of data to provide information on the associations of interest are not always available. Second, MR studies have potential limitations in the statistical power of the study design, which depends on several aspects, such as the proportion of variance in the exposure explained by the genetic instrumental variable and the magnitude of the causal association between exposure and outcome (Brion et al., 2013; Burgessm, 2014). Besides, in recent years, artificial intelligence has been explored to improve the diagnosis of PCa, including radiological and histological diagnoses (Ström et al., 2020; Mata et al., 2021). Ramírez-Mena et al. (2023) combined gene expression and AI for the detection and screening of PCa and found it can decrease the misclassification rates of anatomopathological analysis, thus reducing the need for repeated biopsies. Their algorithm may be applied to urine or blood samples rather than traditional living tissue, which could be a part of the liquid biopsy strategy in PCa in the future. To the best of our knowledge, AI has not been applied in the calculation of causal association between the gut microbiota and the risk of PCa, and we believe the potential causal relationship indicated by MR approaches can provide some references for further studies that focus on the underlying mechanisms.

There are some strengths and limitations in this study. The current study refined the taxonomic units of gut microbiota to the species level and explored their relationship with the risk of PCa, which may be more comprehensive compared to the previous studies. Nevertheless, there were two major limitations in this study. Our study data were extracted from the MiBioGen consortium, DMP, and FinnGen consortium, which may cause the selection bias. Furthermore, these databases only contain the European population, so the potential causal relationship between gut microbiota and the risk of PCa is limited, and whether the results are generalizable to other populations requires further studies to clarify. Another limitation was that the locations of the SNPs we used (whether they are present in the enhancer/promoter/coding region of the genome) are not available, which limited the explanation for the mechanisms of the potential causal association between gut microbiota and PCa.

## Conclusion

Our study suggested a potential causal relationship between gut microbiota and the risk of PCa. Further studies are warranted to elucidate the causal association and specific underlying mechanisms of gut microbiota and the development of PCa.

## Data availability statement

Publicly available datasets were analyzed in this study. This data can be found here: the GWAS Catalog, https://www.ebi.ac.uk/gwas/.

## Author contributions

QX and BH designed the study, collected, analyzed, and interpreted the data. QX wrote the manuscript. BH critically reviewed, edited, and approved the manuscript. Both authors read and approved the final manuscript.
